# *rac*-2-Hy­droxy-2-(2-oxocyclo­pent­yl)-1*H*-indene-1,3(2*H*)-dione

**DOI:** 10.1107/S1600536810042856

**Published:** 2010-10-30

**Authors:** J. Kalyana Sundar, S. Maharani, R. Ranjith Kumar, S. Natarajan, J. Suresh, P. L. Nilantha Lakshman

**Affiliations:** a Department of Physics, Madurai Kamaraj University, Madurai 625 021, India; b Department of Organic Chemistry, School of Chemistry, Madurai Kamaraj University, Madurai 625 021, India; c Department of Physics, The Madura College, Madurai 625 011, India; d Department of Food Science and Technology, University of Ruhuna, Mapalana, Kamburupitiya 81100, Sri Lanka

## Abstract

In the title compound, C_14_H_12_O_4_, the indene unit is essentially planar [r.m.s. deviation = 0.0309 (1) Å] and the cyclo­penta­none ring adopts a twist form. In the crystal, mol­ecules are joined *via* pairs of O—H⋯O hydrogen bonds into centrosymmetric dimers.

## Related literature

For a similar structure, see: Penthala *et al.* (2009 ▶).
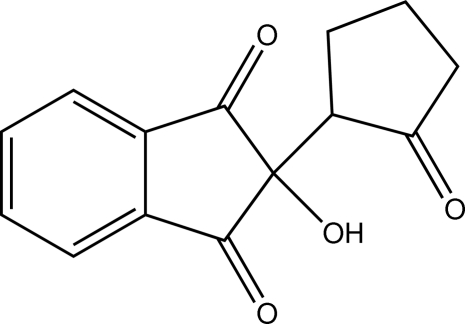


## Experimental

### 

#### Crystal data

C_14_H_12_O_4_
*M* *_r_* = 244.24
Triclinic, 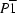

*a* = 8.044 (3) Å
*b* = 8.404 (4) Å
*c* = 10.239 (3) Å
α = 66.95 (3)°
β = 74.36 (2)°
γ = 68.50 (3)°
*V* = 586.1 (4) Å^3^
*Z* = 2
Mo *K*α radiation
μ = 0.10 mm^−1^
*T* = 293 K
0.26 × 0.22 × 0.19 mm

#### Data collection

Nonius MACH3 diffractometer
Absorption correction: ψ scan (North *et al.*, 1968 ▶) *T*_min_ = 0.974, *T*_max_ = 0.981
2534 measured reflections
2054 independent reflections
1886 reflections with *I* > 2σ(*I*)
*R*_int_ = 0.016
3 standard reflections every 60 min  intensity decay: none

#### Refinement

*R*[*F* ^2^ > 2σ(*F* ^2^)] = 0.038
*wR*(*F* ^2^) = 0.104
*S* = 1.07
2054 reflections
164 parameters
H-atom parameters constrained
Δρ_max_ = 0.25 e Å^−3^
Δρ_min_ = −0.23 e Å^−3^

### 

Data collection: *CAD-4 EXPRESS* (Enraf–Nonius, 1994 ▶); cell refinement: *CAD-4 EXPRESS*; data reduction: *XCAD4* (Harms & Wocadlo, 1996 ▶); program(s) used to solve structure: *SHELXS97* (Sheldrick, 2008 ▶); program(s) used to refine structure: *SHELXL97* (Sheldrick, 2008 ▶); molecular graphics: *PLATON* (Spek, 2009 ▶); software used to prepare material for publication: *SHELXL97*.

## Supplementary Material

Crystal structure: contains datablocks global, I. DOI: 10.1107/S1600536810042856/gk2301sup1.cif

Structure factors: contains datablocks I. DOI: 10.1107/S1600536810042856/gk2301Isup2.hkl

Additional supplementary materials:  crystallographic information; 3D view; checkCIF report

## Figures and Tables

**Table 1:** Hydrogen-bond geometry (Å, °)

*D*—H⋯*A*	*D*—H	H⋯*A*	*D*⋯*A*	*D*—H⋯*A*
O2—H2⋯O4^i^	0.82	2.09	2.791 (2)	143

Symmetry code: (i) 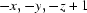.

**Fig. 1.**
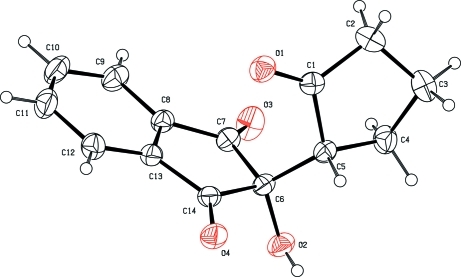
The molecular structure of the title compound showing 30% probability displacement ellipsoids and the atom-numbering scheme.

**Fig. 2.**
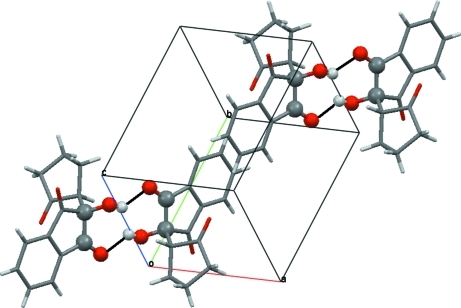
The packing diagram of the title compound.

## supplementary crystallographic information

### Comment

Ninhydrin derivatives constitute a versatile class of compounds with profound biological activities such as antibacterial, anticonvulsant, anticancer and anti-inflammatory activities. The present work constitutes the synthesis of various ninhydrin derivatives which are being tested for anti-tubercular and other biological activities.

### Experimental

A mixture of cyclopentanone (0.5 g, 0.006 mol) and ninhydrin (1.05 g, 0.006 mol) in methanol (10 ml) was heated under reflux for 4 h in the presence of solid sodium ethoxide (0.4 g, 0.006 mol). After completion of the reaction, as was evident from TLC, the reaction mixture was poured into crushed ice, extracted with dichloromethane and subjected to column chromatographic purification using petroleum ether:ethyl acetate mixture (90:10 *v*/*v*) to obtain the product in 50% yield. The compound was further recrystallized from ethyl acetate to obtain suitable crystals for X-ray studies (m.p. 543 K)

### Refinement

All H atoms were placed at calculated positions and allowed to ride on their carrier atoms with C—H = 0.93–0.97 Å, *U*_iso_ = 1.2*U*_eq_(C) and O—H = 0.82 Å, and *U*_iso_ = 1.5*U*_eq_(O) .

### Crystal data

**Table:**

C_14_H_12_O_4_	*Z* = 2
*M**_r_* = 244.24	*F*(000) = 256
Triclinic, *P*1	*D*_x_ = 1.384 Mg m^−^^3^
Hall symbol: -P 1	Mo *K*α radiation, λ = 0.71069 Å
*a* = 8.044 (3) Å	Cell parameters from 25 reflections
*b* = 8.404 (4) Å	θ = 2–25°
*c* = 10.239 (3) Å	µ = 0.10 mm^−^^1^
α = 66.95 (3)°	*T* = 293 K
β = 74.36 (2)°	Block, colourless
γ = 68.50 (3)°	0.26 × 0.22 × 0.19 mm
*V* = 586.1 (4) Å^3^	

### Data collection

**Table:**

Nonius MACH3 diffractometer	1886 reflections with *I* > 2σ(*I*)
Radiation source: fine-focus sealed tube	*R*_int_ = 0.016
graphite	θ_max_ = 25.0°, θ_min_ = 2.2°
ω–2θ scans	*h* = −1→9
Absorption correction: ψ scan (North *et al.*, 1968)	*k* = −9→9
*T*_min_ = 0.974, *T*_max_ = 0.981	*l* = −11→12
2534 measured reflections	3 standard reflections every 60 min
2054 independent reflections	intensity decay: none

### Refinement

**Table:**

Refinement on *F*^2^	Secondary atom site location: difference Fourier map
Least-squares matrix: full	Hydrogen site location: inferred from neighbouring sites
*R*[*F*^2^ > 2σ(*F*^2^)] = 0.038	H-atom parameters constrained
*wR*(*F*^2^) = 0.104	*w* = 1/[σ^2^(*F*_o_^2^) + (0.0487*P*)^2^ + 0.2034*P*] where *P* = (*F*_o_^2^ + 2*F*_c_^2^)/3
*S* = 1.07	(Δ/σ)_max_ < 0.001
2054 reflections	Δρ_max_ = 0.25 e Å^−^^3^
164 parameters	Δρ_min_ = −0.23 e Å^−^^3^
0 restraints	Extinction correction: *SHELXL97* (Sheldrick, 2008), Fc^*^=kFc[1+0.001xFc^2^λ^3^/sin(2θ)]^-1/4^
Primary atom site location: structure-invariant direct methods	Extinction coefficient: 0.059 (7)

### Special details

**Table:**

Geometry. All e.s.d.'s (except the e.s.d. in the dihedral angle between two l.s. planes) are estimated using the full covariance matrix. The cell e.s.d.'s are taken into account individually in the estimation of e.s.d.'s in distances, angles and torsion angles; correlations between e.s.d.'s in cell parameters are only used when they are defined by crystal symmetry. An approximate (isotropic) treatment of cell e.s.d.'s is used for estimating e.s.d.'s involving l.s. planes.
Refinement. Refinement of *F*^2^ against ALL reflections. The weighted *R*-factor *wR* and goodness of fit *S* are based on *F*^2^, conventional *R*-factors *R* are based on *F*, with *F* set to zero for negative *F*^2^. The threshold expression of *F*^2^ > σ(*F*^2^) is used only for calculating *R*-factors(gt) *etc*. and is not relevant to the choice of reflections for refinement. *R*-factors based on *F*^2^ are statistically about twice as large as those based on *F*, and *R*- factors based on ALL data will be even larger.

### Fractional atomic coordinates and isotropic or equivalent isotropic displacement parameters (Å^2^)

**Table:**

	*x*	*y*	*z*	*U*_iso_*/*U*_eq_	
C1	0.4901 (2)	0.0143 (2)	0.33717 (14)	0.0367 (4)	
C2	0.6584 (2)	−0.1372 (2)	0.32374 (19)	0.0505 (4)	
H2A	0.7411	−0.1548	0.3852	0.061*	
H2B	0.7191	−0.1129	0.2254	0.061*	
C3	0.5929 (3)	−0.3013 (2)	0.3708 (2)	0.0551 (5)	
H3A	0.5821	−0.3580	0.4742	0.066*	
H3B	0.6745	−0.3896	0.3258	0.066*	
C4	0.4084 (2)	−0.2245 (2)	0.3207 (2)	0.0494 (4)	
H4A	0.3353	−0.3064	0.3719	0.059*	
H4B	0.4206	−0.2008	0.2184	0.059*	
C5	0.3268 (2)	−0.04868 (19)	0.35711 (15)	0.0367 (4)	
H5	0.2797	−0.0818	0.4598	0.044*	
C6	0.17097 (19)	0.09543 (19)	0.27852 (15)	0.0353 (3)	
C7	0.2311 (2)	0.1817 (2)	0.11679 (15)	0.0387 (4)	
C8	0.2136 (2)	0.3728 (2)	0.09029 (16)	0.0379 (4)	
C9	0.2631 (2)	0.5004 (2)	−0.03520 (18)	0.0502 (4)	
H9	0.3169	0.4710	−0.1186	0.060*	
C10	0.2304 (3)	0.6721 (2)	−0.0329 (2)	0.0573 (5)	
H10	0.2618	0.7600	−0.1163	0.069*	
C11	0.1513 (3)	0.7168 (2)	0.0916 (2)	0.0561 (5)	
H11	0.1308	0.8338	0.0902	0.067*	
C12	0.1028 (2)	0.5900 (2)	0.21723 (19)	0.0470 (4)	
H12	0.0508	0.6193	0.3008	0.056*	
C13	0.13396 (19)	0.4179 (2)	0.21488 (16)	0.0366 (3)	
C14	0.09696 (19)	0.2590 (2)	0.33215 (15)	0.0362 (3)	
O1	0.48152 (16)	0.16243 (15)	0.33474 (12)	0.0484 (3)	
O2	0.03172 (15)	0.02227 (15)	0.29221 (13)	0.0487 (3)	
H2	−0.0080	−0.0170	0.3773	0.073*	
O3	0.2801 (2)	0.10548 (17)	0.02872 (13)	0.0602 (4)	
O4	0.01604 (16)	0.25544 (16)	0.45179 (12)	0.0502 (3)	

### Atomic displacement parameters (Å^2^)

**Table:**

	*U*^11^	*U*^22^	*U*^33^	*U*^12^	*U*^13^	*U*^23^
C1	0.0390 (8)	0.0451 (9)	0.0268 (7)	−0.0147 (7)	−0.0051 (6)	−0.0103 (6)
C2	0.0385 (9)	0.0636 (11)	0.0529 (10)	−0.0090 (8)	−0.0067 (7)	−0.0282 (9)
C3	0.0563 (11)	0.0472 (10)	0.0590 (11)	0.0008 (8)	−0.0178 (8)	−0.0231 (8)
C4	0.0534 (10)	0.0382 (9)	0.0599 (10)	−0.0117 (7)	−0.0158 (8)	−0.0162 (7)
C5	0.0392 (8)	0.0369 (8)	0.0330 (7)	−0.0144 (6)	−0.0043 (6)	−0.0082 (6)
C6	0.0368 (8)	0.0355 (8)	0.0349 (7)	−0.0164 (6)	−0.0048 (6)	−0.0077 (6)
C7	0.0419 (8)	0.0409 (8)	0.0338 (8)	−0.0138 (6)	−0.0051 (6)	−0.0117 (6)
C8	0.0378 (8)	0.0392 (8)	0.0345 (7)	−0.0138 (6)	−0.0071 (6)	−0.0064 (6)
C9	0.0581 (10)	0.0529 (10)	0.0353 (8)	−0.0246 (8)	−0.0039 (7)	−0.0043 (7)
C10	0.0668 (12)	0.0465 (10)	0.0520 (10)	−0.0299 (9)	−0.0110 (9)	0.0044 (8)
C11	0.0639 (11)	0.0356 (9)	0.0691 (12)	−0.0193 (8)	−0.0185 (9)	−0.0073 (8)
C12	0.0483 (9)	0.0397 (9)	0.0527 (10)	−0.0113 (7)	−0.0083 (7)	−0.0158 (7)
C13	0.0335 (7)	0.0367 (8)	0.0372 (8)	−0.0108 (6)	−0.0057 (6)	−0.0087 (6)
C14	0.0312 (7)	0.0400 (8)	0.0343 (8)	−0.0118 (6)	−0.0028 (6)	−0.0091 (6)
O1	0.0516 (7)	0.0463 (7)	0.0537 (7)	−0.0207 (5)	−0.0116 (5)	−0.0145 (5)
O2	0.0464 (7)	0.0514 (7)	0.0525 (7)	−0.0268 (5)	−0.0123 (5)	−0.0066 (5)
O3	0.0894 (10)	0.0528 (7)	0.0402 (6)	−0.0226 (7)	−0.0035 (6)	−0.0195 (6)
O4	0.0528 (7)	0.0511 (7)	0.0383 (6)	−0.0173 (5)	0.0087 (5)	−0.0143 (5)

### Geometric parameters (Å, °)

**Table:**

C1—O1	1.2116 (19)	C6—C7	1.545 (2)
C1—C2	1.499 (2)	C7—O3	1.2059 (19)
C1—C5	1.526 (2)	C7—C8	1.479 (2)
C2—C3	1.512 (3)	C8—C9	1.384 (2)
C2—H2A	0.9700	C8—C13	1.393 (2)
C2—H2B	0.9700	C9—C10	1.378 (3)
C3—C4	1.528 (3)	C9—H9	0.9300
C3—H3A	0.9700	C10—C11	1.391 (3)
C3—H3B	0.9700	C10—H10	0.9300
C4—C5	1.531 (2)	C11—C12	1.379 (3)
C4—H4A	0.9700	C11—H11	0.9300
C4—H4B	0.9700	C12—C13	1.383 (2)
C5—C6	1.534 (2)	C12—H12	0.9300
C5—H5	0.9800	C13—C14	1.469 (2)
C6—O2	1.4160 (18)	C14—O4	1.2169 (18)
C6—C14	1.536 (2)	O2—H2	0.8200
			
O1—C1—C2	126.74 (15)	C5—C6—C14	110.99 (12)
O1—C1—C5	124.60 (14)	O2—C6—C7	107.82 (12)
C2—C1—C5	108.66 (13)	C5—C6—C7	113.02 (12)
C1—C2—C3	104.51 (14)	C14—C6—C7	102.49 (12)
C1—C2—H2A	110.9	O3—C7—C8	126.80 (14)
C3—C2—H2A	110.9	O3—C7—C6	125.30 (14)
C1—C2—H2B	110.9	C8—C7—C6	107.89 (13)
C3—C2—H2B	110.9	C9—C8—C13	120.56 (15)
H2A—C2—H2B	108.9	C9—C8—C7	129.09 (15)
C2—C3—C4	103.75 (14)	C13—C8—C7	110.35 (13)
C2—C3—H3A	111.0	C10—C9—C8	118.04 (17)
C4—C3—H3A	111.0	C10—C9—H9	121.0
C2—C3—H3B	111.0	C8—C9—H9	121.0
C4—C3—H3B	111.0	C9—C10—C11	121.33 (16)
H3A—C3—H3B	109.0	C9—C10—H10	119.3
C3—C4—C5	102.74 (13)	C11—C10—H10	119.3
C3—C4—H4A	111.2	C12—C11—C10	120.90 (17)
C5—C4—H4A	111.2	C12—C11—H11	119.6
C3—C4—H4B	111.2	C10—C11—H11	119.6
C5—C4—H4B	111.2	C11—C12—C13	117.90 (17)
H4A—C4—H4B	109.1	C11—C12—H12	121.1
C1—C5—C4	103.78 (13)	C13—C12—H12	121.1
C1—C5—C6	114.77 (12)	C12—C13—C8	121.28 (14)
C4—C5—C6	117.09 (13)	C12—C13—C14	129.02 (15)
C1—C5—H5	106.9	C8—C13—C14	109.69 (14)
C4—C5—H5	106.9	O4—C14—C13	126.49 (15)
C6—C5—H5	106.9	O4—C14—C6	124.45 (13)
O2—C6—C5	111.26 (12)	C13—C14—C6	109.03 (12)
O2—C6—C14	110.92 (12)	C6—O2—H2	109.5
			
O1—C1—C2—C3	166.12 (15)	O3—C7—C8—C13	173.24 (16)
C5—C1—C2—C3	−13.03 (17)	C6—C7—C8—C13	−5.46 (17)
C1—C2—C3—C4	33.50 (18)	C13—C8—C9—C10	−0.3 (2)
C2—C3—C4—C5	−41.27 (18)	C7—C8—C9—C10	179.19 (16)
O1—C1—C5—C4	168.45 (14)	C8—C9—C10—C11	0.5 (3)
C2—C1—C5—C4	−12.37 (16)	C9—C10—C11—C12	−0.1 (3)
O1—C1—C5—C6	39.4 (2)	C10—C11—C12—C13	−0.5 (3)
C2—C1—C5—C6	−141.39 (14)	C11—C12—C13—C8	0.7 (2)
C3—C4—C5—C1	32.54 (16)	C11—C12—C13—C14	179.53 (15)
C3—C4—C5—C6	160.13 (14)	C9—C8—C13—C12	−0.3 (2)
C1—C5—C6—O2	174.96 (11)	C7—C8—C13—C12	−179.90 (14)
C4—C5—C6—O2	52.92 (18)	C9—C8—C13—C14	−179.32 (14)
C1—C5—C6—C14	−61.01 (16)	C7—C8—C13—C14	1.09 (17)
C4—C5—C6—C14	176.94 (12)	C12—C13—C14—O4	7.0 (3)
C1—C5—C6—C7	53.49 (17)	C8—C13—C14—O4	−174.07 (15)
C4—C5—C6—C7	−68.56 (17)	C12—C13—C14—C6	−175.15 (15)
O2—C6—C7—O3	−54.4 (2)	C8—C13—C14—C6	3.77 (16)
C5—C6—C7—O3	69.0 (2)	O2—C6—C14—O4	56.38 (19)
C14—C6—C7—O3	−171.52 (16)	C5—C6—C14—O4	−67.85 (19)
O2—C6—C7—C8	124.29 (13)	C7—C6—C14—O4	171.22 (14)
C5—C6—C7—C8	−112.31 (14)	O2—C6—C14—C13	−121.51 (13)
C14—C6—C7—C8	7.21 (15)	C5—C6—C14—C13	114.26 (13)
O3—C7—C8—C9	−6.3 (3)	C7—C6—C14—C13	−6.67 (15)
C6—C7—C8—C9	174.99 (15)		

### Hydrogen-bond geometry (Å, °)

**Table:**

*D*—H···*A*	*D*—H	H···*A*	*D*···*A*	*D*—H···*A*
O2—H2···O4^i^	0.82	2.09	2.791 (2)	143
C2—H2A···O4^ii^	0.97	2.60	3.560 (2)	171
C2—H2B···O3^iii^	0.97	2.58	3.423 (2)	146

Symmetry codes: (i) −*x*, −*y*, −*z*+1; (ii) −*x*+1, −*y*, −*z*+1; (iii) −*x*+1, −*y*, −*z*.
